# Genome-Scale Screen for DNA Methylation-Based Detection Markers for Ovarian Cancer

**DOI:** 10.1371/journal.pone.0028141

**Published:** 2011-12-07

**Authors:** Mihaela Campan, Melissa Moffitt, Sahar Houshdaran, Hui Shen, Martin Widschwendter, Günter Daxenbichler, Tiffany Long, Christian Marth, Ite A. Laird-Offringa, Michael F. Press, Louis Dubeau, Kimberly D. Siegmund, Anna H. Wu, Susan Groshen, Uma Chandavarkar, Lynda D. Roman, Andrew Berchuck, Celeste L. Pearce, Peter W. Laird

**Affiliations:** 1 Department of Surgery, Keck School of Medicine, University of Southern California, Los Angeles, California, United States of America; 2 Department of Obstetrics and Gynecology Division of Gynecologic Oncology, Keck School of Medicine, University of Southern California, Los Angeles, California, United States of America; 3 Department of Biochemistry and Molecular Biology, Keck School of Medicine, University of Southern California, Los Angeles, California, United States of America; 4 Department of Gynecological Oncology, Elizabeth Garrett Anderson Institute for Women's Health, University College London, London, United Kingdom; 5 Department of Obstetrics and Gynecology, Innsbruck Medical University, Innsbruck, Austria; 6 Department of Obstetrics and Gynecology Division of Gynecologic Oncology, Duke University Medical Center, Durham, North Carolina, United States of America; 7 Department of Preventive Medicine, Keck School of Medicine, University of Southern California, Los Angeles, California, United States of America; 8 University of Southern California Epigenome Center, University of Southern California, Los Angeles, CA, United States of America; 9 Department of Pathology, Keck School of Medicine, University of Southern California, Los Angeles, California, United States of America; Deutsches Krebsforschungszentrum, Germany

## Abstract

**Background:**

The identification of sensitive biomarkers for the detection of ovarian cancer is of high clinical relevance for early detection and/or monitoring of disease recurrence. We developed a systematic multi-step biomarker discovery and verification strategy to identify candidate DNA methylation markers for the blood-based detection of ovarian cancer.

**Methodology/Principal Findings:**

We used the Illumina Infinium platform to analyze the DNA methylation status of 27,578 CpG sites in 41 ovarian tumors. We employed a marker selection strategy that emphasized sensitivity by requiring consistency of methylation across tumors, while achieving specificity by excluding markers with methylation in control leukocyte or serum DNA. Our verification strategy involved testing the ability of identified markers to monitor disease burden in serially collected serum samples from ovarian cancer patients who had undergone surgical tumor resection compared to CA-125 levels.

We identified one marker, *IFFO1* promoter methylation (IFFO1-M), that is frequently methylated in ovarian tumors and that is rarely detected in the blood of normal controls. When tested in 127 serially collected sera from ovarian cancer patients, IFFO1-M showed post-resection kinetics significantly correlated with serum CA-125 measurements in six out of 16 patients.

**Conclusions/Significance:**

We implemented an effective marker screening and verification strategy, leading to the identification of IFFO1-M as a blood-based candidate marker for sensitive detection of ovarian cancer. Serum levels of IFFO1-M displayed post-resection kinetics consistent with a reflection of disease burden. We anticipate that IFFO1-M and other candidate markers emerging from this marker development pipeline may provide disease detection capabilities that complement existing biomarkers.

## Introduction

Ovarian cancer is the leading cause of gynecological cancer deaths and the fifth leading cause of all cancer-related deaths in women. It has been estimated that one woman in 72 will develop ovarian cancer in her lifetime in the USA, and that one woman in 96 will die of this disease [1]. The five-year overall survival is strongly stage-dependent [2], [3] with rates of 94% for stage I disease and 28% for stage IV disease [1].

Since early stage disease is often asymptomatic, and there is no effective screening strategy, most patients (62%) present with advanced-stage (III and IV) disease, in which the cancer has spread throughout the peritoneal cavity or other organs [1]. More than 85% of patients with advanced disease relapse after cessation of primary therapy, despite an initial good response [4], [5]. It is anticipated that effective methods for detection of asymptomatic ovarian cancer before invasion and metastasis has occurred would substantially reduce the mortality rate for this disease. Sensitive detection methods could also be applied to monitoring disease recurrence after tumor resection with or without adjuvant chemotherapy.

Currently, there is no good biomarker or imaging approach with sufficient sensitivity and specificity for the detection of preclinical ovarian cancer [6]. Two protein-based biomarkers, CA-125 and HE4, have been clinically approved to measure disease burden and to evaluate ovarian cancer treatment [7], [8]. However, these markers are not elevated in all ovarian tumors and do not have sufficient positive predictive value for population-based risk assessment or early detection. Given the limitations of current approaches, there is an urgent need to develop more effective strategies for the detection of preclinical ovarian cancer early enough for treatment to be successful. Since ovarian cancers are heterogeneous, with unknown cells of origin and poorly understood pathogenesis [9] the marker discovery processes should rely on high-throughput technology-based approaches rather than on mechanistic-driven marker discovery strategies. Also, markers for ovarian cancer should be able to detect tumors hundreds of times smaller than the clinically apparent serous cancers typically used to evaluate biomarker performance [10].

Epigenetic biomarkers have recently emerged as alternatives to protein biomarkers for the early detection of cancer [11]–[13], including ovarian cancers [14]–[17]. Aberrant DNA hypermethylation is frequently observed in cancer cells [18]. Cancer patients have elevated levels of free DNA circulating in the bloodstream [19]. Cancer-associated aberrant DNA methylation, originated at least in part in tumor cells, can be detected in serum or plasma DNA of cancer patients [11], [12]. Methylated DNA is chemically and biologically stable, readily detectable in many types of bodily fluids and therefore well suited for blood-based cancer detection [11]–[17]. However, the limited number of DNA methylation markers currently available apply to only a small fraction of ovarian cancers [14] and are non-specific, while the detection technologies lack sensitivity, are largely gel-based, and are non-quantitative [15]–[17]. Recent advances in DNA methylation assay technologies have the potential to increase the DNA methylation marker discovery throughput through the simultaneous analysis of thousands of genomic loci [20], [21] and to allow for ultra sensitive detection of very small amounts of methylated DNA in a quantitative manner [22], [23].

In this study, we conducted a large-scale systematic marker discovery for DNA methylation markers of ovarian cancer that are not present in the blood of women without ovarian cancer. DNA methylation markers have been found to have moderate clinical sensitivity in many prior reports. In considering how to improve the sensitivity of DNA methylation markers, we recognized that the methylation status of normal ovary is irrelevant, as long as normal ovary DNA does not normally leak into the bloodstream and the markers are negative in healthy controls. Therefore, we modified our discovery strategy to focus on a direct comparison of tumor vs. blood, as opposed to tumor vs. normal tissue. In our selection process we emphasized marker sensitivity by requiring consistency of tumor methylation, and marker specificity by excluding markers with methylation in control leukocyte or serum DNA. We identified a promising candidate DNA methylation marker, IFFO1-M, which we tested as a blood-based biomarker in a limited number of case and control sera. To provide evidence that our candidate IFFO1-M marker measures disease burden in the blood, we analyzed the temporal patterns of IFFO1-M levels in serial blood samples drawn before and after resection of the primary tumor, and compared these to a validated marker for disease burden, CA-125. This within-subject comparison allows each patient to serve as her own control, with no variation in genetic background between the serial blood samples. In this study, we report on the quantitative digital analysis of IFFO1-M in serial samples from nine patients, for a total of 127 blood samples.

## Methods

### Ethics Statement

This study was conducted in accordance with the Helsinki human subjects doctrine and was approved by the Institutional Review Boards of the institutes involved in the study: Duke University Medical Center (Durham, USA), Keck School of Medicine of the University of Southern California (Los Angeles, USA), and Innsbruck University Hospital (Innsbruck, Austria). Signed informed consent was obtained from all study participants for the collection of the samples and their subsequent analysis.

### Patients and controls specimen collection and processing

The 41 ovarian tumor samples used in the Infinium-based marker discovery phase of the study were obtained from patients that underwent surgery at two institutions, Duke University Medical Center (30 samples) and University of Southern California Medical Center (11 samples). All tumor samples were obtained from patients who provided written informed consent, which was approved by the Institutional Review Boards of the respective institutions. Among the tumor samples collected, there was one mixed (clear cell and endometrioid), three clear cell, four mucinous, four endometrioid, and 32 serous epithelial ovarian carcinoma samples (Table S1). Tumor tissues were flash-frozen in liquid nitrogen and stored at −80°C until processed. Peripheral blood leukocyte (PBL) and plasma samples used in the discovery and verification stages were obtained from 10 healthy post-menopausal women whose bloods were commercially purchased (HemaCare Corporation). Plasma was isolated from blood collected in tubes containing EDTA. The tubes were spun for 10 min at 300 *g* at 4°C. Without removing the plasma from the tube after the first centrifugation, we spun the tubes for an additional 10 min at 1,600 *g* at 4°C. The separated plasma was transferred to microcentrifuge tubes and spun again at 16,000 *g* for 10 min at 4°C. The supernatant was collected and stored at −80°C until ready to use. The thin peripheral blood leukocytes layer that sedimented above the red blood cells was collected and stored −80°C until ready to use for DNA extraction.

DNA was extracted from tissues using standard protocols. DNA was extracted from the PBL samples using the QIAamp® DNA Blood kit (Qiagen), while free DNA from plasma and sera was extracted using the QIAamp® UltraSens Virus Kit (Qiagen). In both experiments, DNA was extracted following the manufacturer's instructions.

The blood samples used in the longitudinal analyses were collected from 16 patients treated for ovarian cancer between 1992–2000 at the Department of Obstetrics and Gynecology, Innsbruck University Hospital (Innsbruck, Austria) in compliance with and approved by the Innsbruck University Institutional Review Board. The clinical and pathological characteristics of these patients are listed in Table S3. The first blood samples drawn, referred to as baseline samples, were obtained before the surgery for eleven of the patients and several days after the surgery for five patients (see complete information in Table S4). Additional blood was collected from all patients at each follow-up visit for periods of times ranging from 37 to 246 weeks (Table S4). For serum isolation, blood was allowed to coagulate for 1–4 hours at room temperature (RT) and centrifuged for 10 minutes at 2000 *g* at RT. Serum was isolated from the clot, aliquoted into microcentrifuge tubes, and stored at −80°C until analysis. Control sera from eight healthy women were commercially purchased (Innovative Research). Free circulating DNA was isolated from the patients and controls sera using the QIAamp® UltraSens Virus Kit (Qiagen) following the manufacturer's instructions. Levels of CA-125 were determined by a micro-particle enzyme-immunoassay (MEIA) using the IMX analyzer (Abbott Laboratories).

### DNA methylation analysis

All DNA specimens were subjected to bisulfite modification using the EZ DNA Methylation Kit (Zymo Research) according to the manufacturer's instructions. For the Infinium-based analysis, 1 µg genomic DNA from each sample was bisulfite converted in 96 well plate format using the EZ96 DNA methylation kit (Zymo Research). The quality and quantity of the bisulfite-converted DNA, as well as the completeness of the bisulfite conversion, were assessed using a panel of quality control reactions as previously described [24]. Following the conversion, the modified DNA was eluted in 18 µl elution buffer supplied with the kit, and 5 µl of each sample was used in the Illumina Infinium DNA methylation assay as specified by the manufacturer.

For MethyLight analysis, 1 µg genomic DNA from each tumor and PBL sample was treated with bisulfite using the Zymo EZ DNA methylation kit (Zymo Research). Similarly, the Zymo EZ DNA methylation kit was used to bisulfite convert the DNA extracted from plasma or sera samples. In general 1 ml of plasma or sera was processed in one column of the Zymo kit. After purification, all bisulfite modified DNA samples were eluted in 10 µl of elution buffer and further diluted as follows: the PBL-DNA was diluted to a final concentration of 0.5 ng/µl. The tumor DNA was diluted based on the cycle threshold (Ct) of an ALU-based MethyLight reaction. Only DNAs with a Ct less than 21 for this ALU-based reaction were used in any of the subsequent analyses [24]. The plasma DNA was diluted such that every 1 µl of modified DNA represented 10 µl of the initial volume of plasma or sera used. For each MethyLight reaction 10 µl of the diluted tumor, PBL or plasma bisulfite converted DNA were used. For Digital MethyLight analysis, the entire amount of DNA extracted from 1 ml of serum was bisulfite converted, and the samples were diluted such that every 1 µl of each bisulfite-converted DNA sample represented 1 µl of the initial serum volume used. In each Digital MethyLight analysis, 100 µl of the diluted DNA was used.

The Infinium analysis was performed in the USC Epigenome center using the HumanMethylation27 BeadArray (Illumina). The protocols and the probe information are available at www.illumina.com. The results of the Infinium assay were compiled for each locus using Illumina BeadStudio software (Illumina) and are reported as beta (β) values which are DNA methylation scores ranging from 0 to 1 that reflect the fractional DNA methylation level of a single CpG site [20]. The Infinium analysis results are available for download at Gene Expression Omnibus (GEO) data repository at the National Center for Biotechnology Information (NCBI) under the accession number GSE26989 (www.ncbi.nlm.nih.gov/geo/query/acc.cgi?acc=GSE26989).

The MethyLight assay and data analysis were performed as previously described [22], [25]. The primers and probes for these analyses are listed in the Table S2. The Digital MethyLight assay and data analysis were performed as previously described [23] with the difference that the PCR reactions were performed in a volume of 10 µl instead of 30 µl.

### Marker selection

Infinium probes that failed in any of the samples were excluded from the analysis. Probes associated with single nucleotide polymorphisms (SNPs), identified using the NCBI dbSNP builds 126 and 128, or repetitive elements identified by RepeatMasker were also excluded. The two PBL samples used in the analysis were run in duplicate on the Infinium DNA methylation platform and the averaged β values for each sample were used for marker filtering. We eliminated any probes with a β value higher or equal to 0.2 in any of the two averaged PBL samples. We identified the tumor sample with the lowest DNA methylation value (T_L_) and the PBL sample with the highest DNA methylation value (PBL_H_) for each probe and we calculated the difference between the ß values of these two samples (T_L_- PBL_H_). The probes were ranked based on this difference, and the probes for which the difference was less or equal to 0 were eliminated.

### Verification of the 15 top-ranked markers using The Cancer Genome Atlas (TCGA) data

The ß values of the 15 top-ranked markers were retrieved from the publically available DNA methylation dataset for serous ovarian cancers posted on the TCGA Data Portal (htpp://tcga-data.nci.nih.gov/tcga). We compared the distribution of Infinium-generated β values of these 15 markers in all the 41 ovarian cancer samples or just the 29 serous ovarian cancers of this study to those obtained from 284 serous ovarian cancer samples in the TCGA study and a total of 10 normal PBL samples using dot-diagrams from the GraphPad Prism Software (GraphPad Software Inc.).

### Statistical analysis

The ability of IFFO1-M to discriminate between case and control samples was assessed by plotting the receiver operating characteristics curve, which associates the true positive rate (sensitivity) to the false positive rate (1-specificity) and by computing area under the curve (AUC). The 95% Confidence Interval (CI) of the AUC was computed with 2000 bootstrap samples using the pROC R package [26]. Pearson product-moment correlation coefficient (Pearson's r) was calculated to measure the degree of correlation between the IFFO1-M DNA methylation levels and the CA-125 levels in each of the nine patients. Statistical test against the null hypothesis that r = 0 was performed and a *p* value cutoff of 0.05 was used to declare significance. All statistical analyses were performed using the R 2.13 software.

## Results

### DNA methylation-based marker discovery pipeline

**Figure 1:**
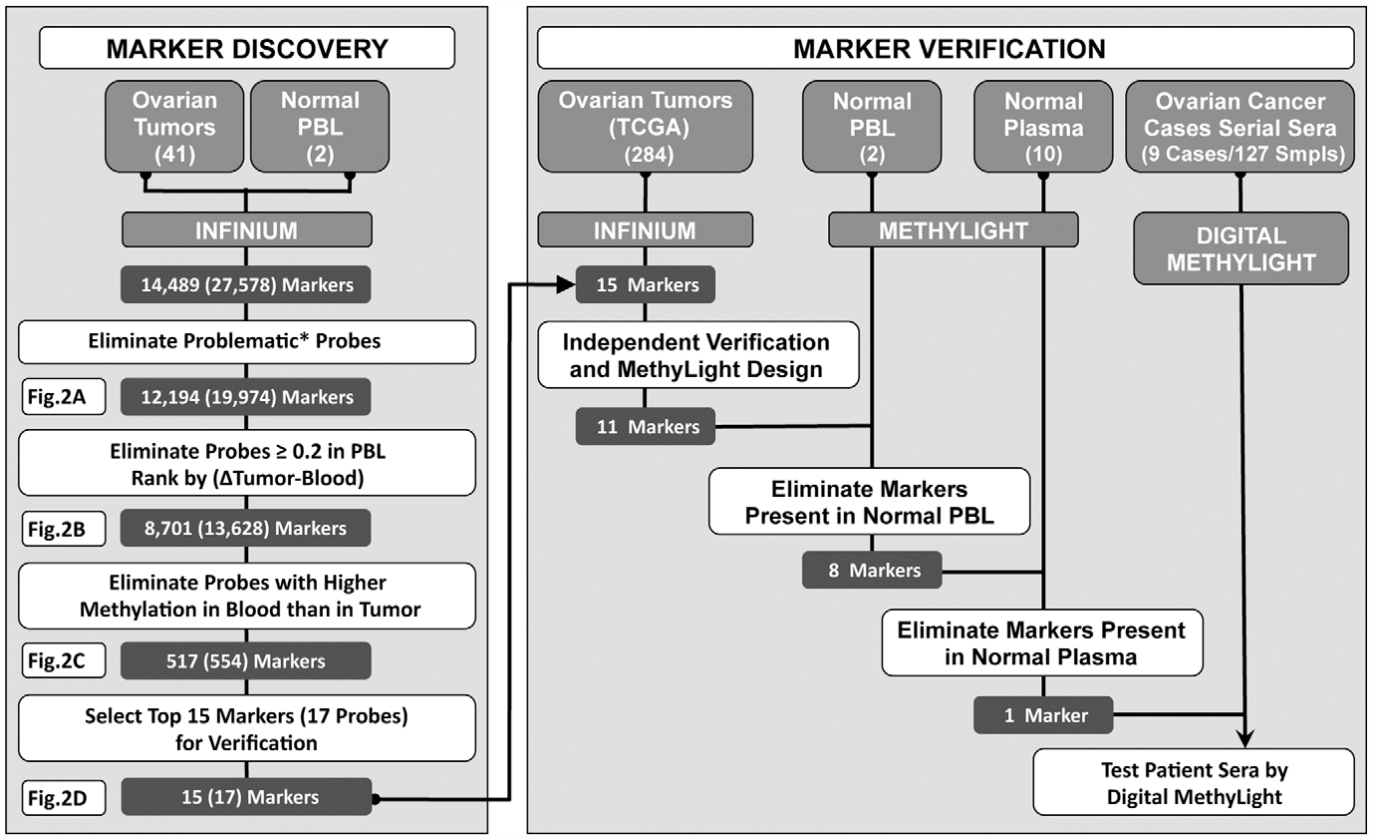
Schematic representation of the ovarian cancer marker discovery and verification pipeline. The Infinium platform was used to screen 27,578 probes representing 14,489 individual gene loci. We used a systematic stepwise approach to eliminate probes that failed in any of the samples, probes that contained SNPs or repeat sequences, or probes with a beta value higher than 0.2 in any of the PBL samples. The remaining probes were ranked based on their difference between tumors and blood (see Materials and Methods), and the probes with higher DNA methylation in PBL than in any of the tumor samples were eliminated. The top 15 from the remaining 517 markers were transitioned to the MethyLight platform for further verification. All 15 markers passed an independent verification test performed on publically available TCGA ovarian cancer dataset and additional PBL samples. Three markers failed due to incompatibility issues related to the MethyLight platform, while another ten failed because they were methylated in normal PBL DNA (3 markers) or normal plasma (7 markers). Only one marker, *IFFO1*, was selected for further verification on patient samples using Digital MethyLight. (The asterisk indicates probes that failed in any of the samples, as well as those that included SNPs and repeat sequences).

We devised a comprehensive and systematic strategy to identify and evaluate blood-based DNA methylation markers for ovarian cancer that are only present in the blood from individuals afflicted with the disease. Figure 1 illustrates the steps we undertook to achieve this goal. Each of these steps is described in more detail in the subsequent sections.

### Screening for and selection of DNA methylation markers for ovarian cancer

**Figure 2:**
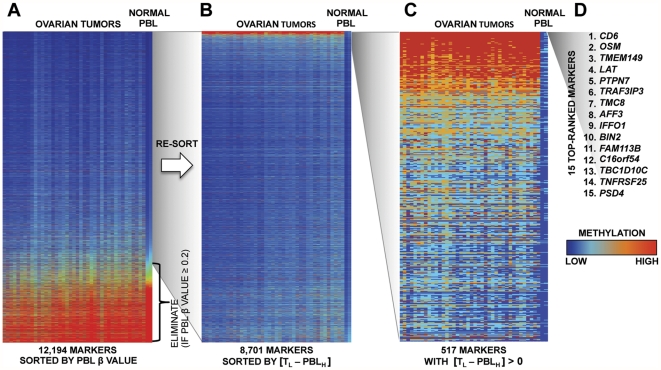
Heat map representation of the marker selection process. A, the 12,194 markers remaining after the elimination of the probes that failed in any of the samples, and of the probes containing SNPs or repetitive elements. Markers are ranked in an ascending order based on the mean DNA methylation β value of the two PBL samples. B, the 8,701 markers remaining after eliminating probes with DNA methylation β values≥0.2 in any of the two PBL samples. Probes were ranked in a descending order based on the difference in DNA methylation between the tumor with the lowest β value (T_L_) and the PBL sample with the highest β value (PBL_H_). C, the 517 markers with higher DNA methylation values in any of the tumor than in any of the PBL samples. The markers are ranked in a descending order based on the difference between the tumors and the PBL DNA methylation values. D, the top-ranked 15 markers that were transitioned to the MethyLight platform for further verification.

We first conducted a large-scale DNA methylation analysis of 41 ovarian cancer samples (Table S1) and two PBL samples from disease-free postmenopausal women. We used the Infinium DNA methylation BeadArray that simultaneously interrogates the DNA methylation status of 27,578 probes spanning 14,489 unique genetic loci. We began the marker selection process by filtering out all probes that failed (detection p-value>0.05) in any of the samples, as well as probes containing single-nucleotide polymorphisms (SNPs) [27], and repeat sequences (Fig. 1 and 2A). We next eliminated all the probes with high DNA methylation levels in PBL (β>0.2 in any one of the PBL sample) (Fig. 1). The remaining 13,628 probes (8701 unique genes) were ranked in a descending order based on an algorithm that calculates the difference between the least methylated ovarian tumor sample and the most methylated blood sample for each probe (Fig. 1 and 2B) (Materials and Methods). The 554 probes (517 unique genes) with higher DNA methylation in any of the ovarian tumors compared to the two normal blood samples were retained for future evaluation (Fig. 1 and 2C). We next performed confirmatory analyses with the top 15-ranked markers (Fig. 1 and 2D). The choice of testing only this limited number of markers was motivated by cost and patient sample availability constrains.

### Independent assessment of reproducibility of the top 15-ranked makers

**Figure 3:**
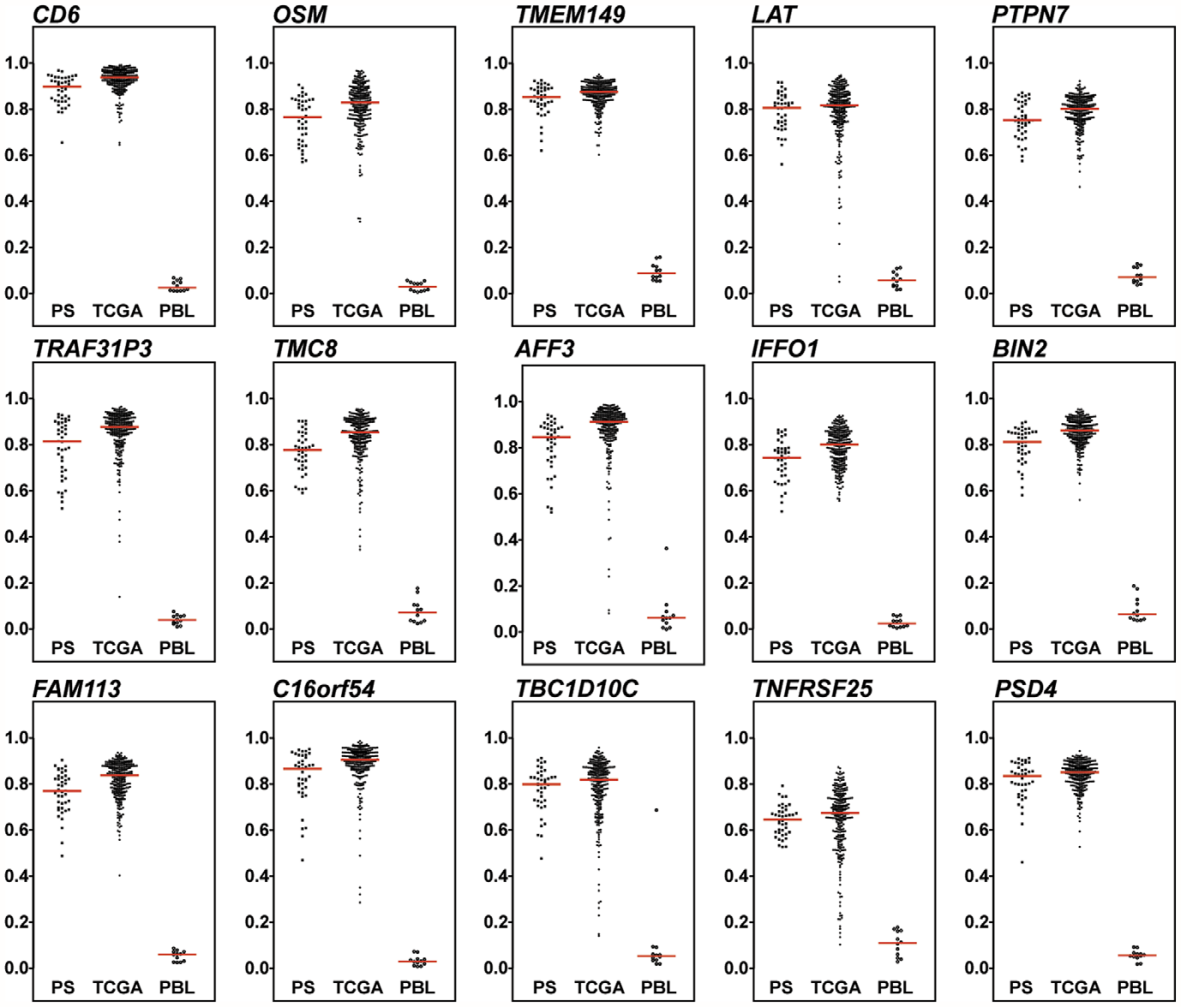
Dot plot display of the top 15-ranked marker distribution in two independent data sets of ovarian cancer samples and ten normal PBL samples. The Infinium-derived β values (Y-axis) for the top 15-ranked markers were compared in the present study (PS) data set (41 ovarian cancers of mixed subtypes), the TCGA data set (284 serous ovarian cancers) and ten normal PBL samples. The horizontal lines represent the median values for each group.

Since the number of ovarian cancer samples used in the screening step was relative small, we took advantage of the publicly available TCGA DNA methylation data on serous ovarian cancers [28] to confirm the performance of the 15 top-ranked markers in an independent larger dataset. This analysis was facilitated by the fact that the TCGA data were generated using the same technology as in our study. The distribution of the DNA methylation beta values for all 15 markers in the two tumor datasets (present study (PS) and TCGA) in comparison to a set of ten healthy control PBL samples is shown in Figure 3. The range of DNA methylation values in our experimental set of 41 serous, mucinous, clear-cell, and endometrioid ovarian cancers was similar to that of the 284 serous ovarian cancer samples from TCGA, with both showing much higher DNA methylation levels than in the ten healthy control PBL samples. The results did not differ when the analysis was restricted to the 29 of the 41 ovarian cancer patients with serous histology (data not shown).

### MethyLight assays development and verification in control samples for the 15 top-ranked markers

**Figure 4:**
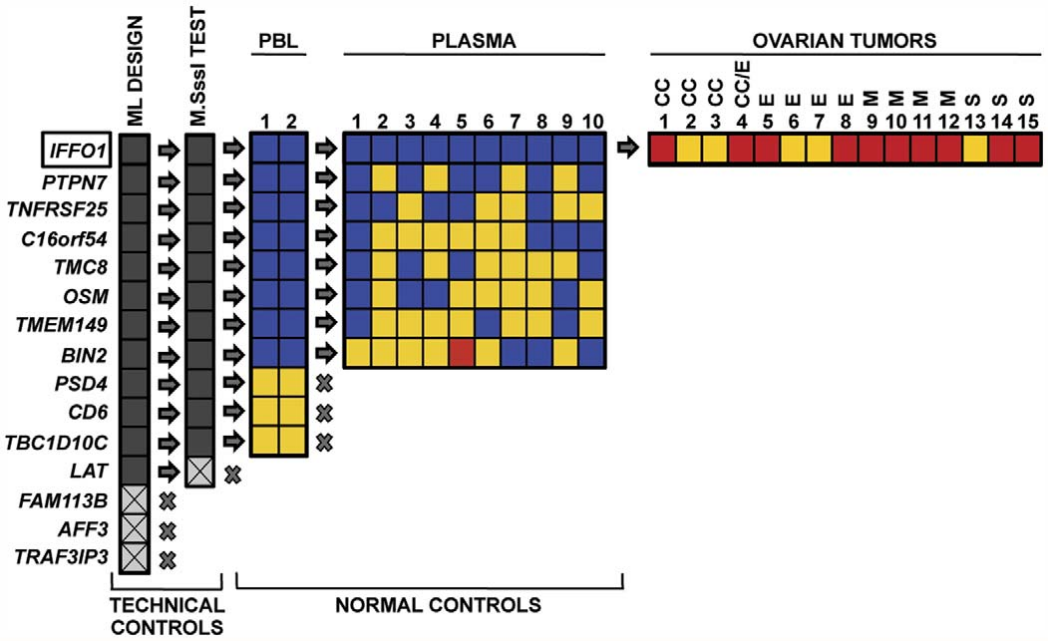
Representation of the verification phase on the MethyLight platform of the top-ranked 15 DNA methylation markers. Technical controls for the MethyLight (ML) platform led to the elimination of four markers (crossed gray boxes) due to design incompatibility, and failure to amplify the *in vitro* methylated DNA positive control for MethyLight reactions (M.SssI test). Eleven markers were tested in normal PBL samples using an excess of PBL DNA (50 ng). Markers with a cycle threshold (Ct) higher than 35 (blue boxes) in the two normal PBL samples were retained and markers with a Ct less than 35 (yellow boxes) were eliminated. MethyLight assays with Ct values<35 indicate appreciably detectable amounts of methylated DNA at these loci. Further testing in normal control plasma samples (100 µl) resulted in the elimination of seven of the eight remaining markers. One remaining marker, *IFFO1*, was tested in 15 ovarian cancers of different histological subtypes. The MethyLight results for the normal plasma control and the ovarian cancer samples are expressed as Percent of Methylated Reference (PMR). Blue boxes represent PMR values less than 10, yellow boxes indicate PMR values between 10 and 50, whereas red boxes signify PMR values higher than 50. The types of ovarian tumors used in the analysis are as follows: clear cell carcinomas (CC), mixed clear cell and endometrioid (CC/E), endometrioid (E), mucinous (M), and serous (S).

We next transitioned the 15 markers to the more sensitive PCR-based DNA methylation detection platforms, MethyLight and Digital MethyLight. Unlike Illumina Infinium DNA methylation probes, which assay the DNA methylation status of a single cytosine, MethyLight-based primers and probe interrogate concordant DNA methylation of several methylated cytosines simultaneously over a short genomic region. Consequently, MethyLight results for a specific genetic locus may differ from those registered by an Illumina Infinium probe at the same location due to the presence of neighboring cytosines and variations in the primers/probe positioning. We were successful in developing MethyLight reactions for 12 of the 15 candidate markers (Table S2). The DNA sequences adjacent to the Infinium targeted cytosines for three of the 15 markers were not suitable for MethyLight design (Fig. 4). An additional marker was eliminated from the pipeline because it failed to amplify *in vitro* methylated (M.SssI-treated) control DNA (Fig. 4). We subjected the remaining 11 markers to a stringent counter screen against excess amounts of PBL DNA (50 ng) from two disease-free postmenopausal women, to exclude DNA methylation markers that would present background problems for the sensitive detection of ovarian DNA methylation markers in blood. This MethyLight-based screen yielded eight markers with very low levels of DNA methylation in PBL (MethyLight cycle thresholds (Ct) higher than 35 in both samples).

These eight markers were subsequently evaluated on concentrated free plasma DNA samples from ten healthy postmenopausal women, yielding only one marker, *IFFO1*, with almost undetectable DNA methylation in all of these control plasma samples (PMRs<5) (Fig. 4). We next tested *IFFO1* on 15 ovarian tumors of various histological subtypes, 14 of which have also been used in the initial screening step (Table S1), and found significant levels of DNA methylation (PMR>20) in all the tumors analyzed (Fig. 4). This is in agreement with the Infinium DNA methylation analysis from the same tumor samples (Fig. 2). This marker was therefore selected for subsequent clinical verification in a set of serum samples collected longitudinally from a separate set of ovarian cancer patients.

### IFFO1 gene promoter DNA methylation (IFFO1-M) marker performance in serum samples

**Figure 5:**
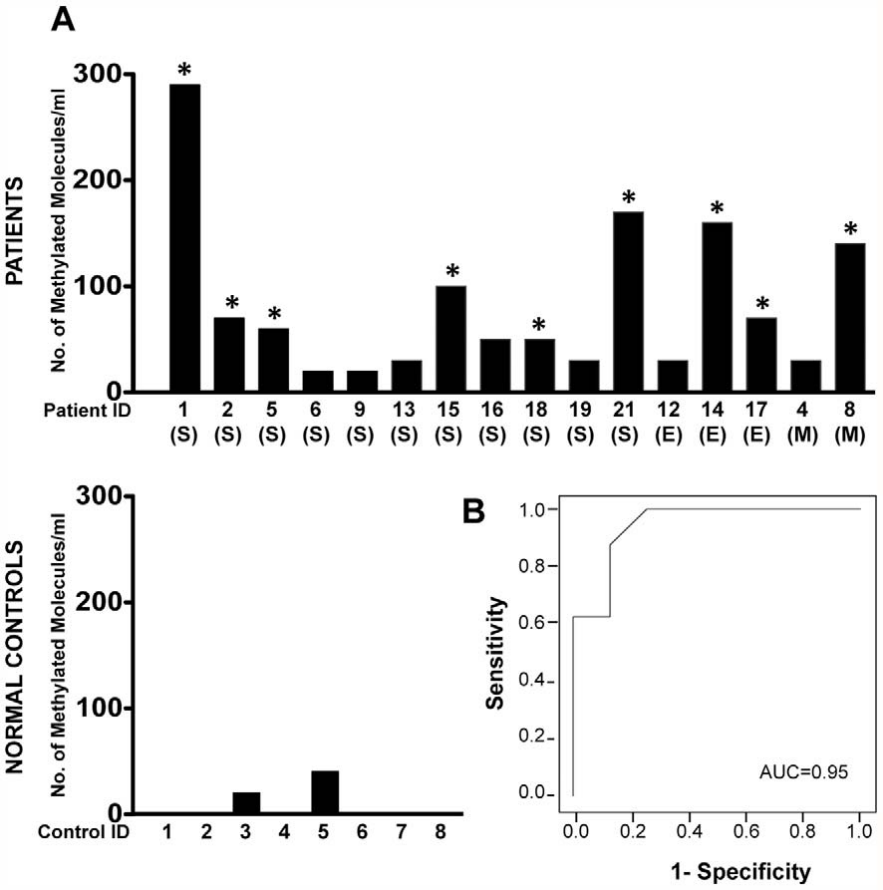
The performance of IFFO1-M marker in the baseline serum samples of ovarian cancer patients and disease-free control women. A, IFFO1-M levels (expressed as the number of IFFO1-M methylated molecules detected in 1 ml sera) in the baseline samples of 16 patients and eight normal controls were determined by Digital MethyLight. The number of molecules in patient #1 is an approximation since counts higher than 15 hits/96-well plate/100 µl tested could reflect the presence of more than one molecule/well. The histological subtype of the tumors is indicated in parenthesis as follows: serous (S), mucinous (M), and endometrioid (E). The asterisks indicate the patient from whom samples were used in the subsequent longitudinal analysis. B, Receiver operating characteristic curve for IFFO1-M. AUC = area under the curve.

We first evaluated the performance of IFFO1-M marker in serum samples obtained from eight healthy older women controls and 16 ovarian cancer patients with advanced (stage III and IV) disease using the highly sensitive and quantitative Digital MethyLight assay [23]. The clinicopathological characteristics of the ovarian cancer patients included in this analysis are summarized in Table S3. The output for Digital MethyLight is measured by counting the individually methylated DNA molecules. We detected the IFFO1-M marker in all patient samples. In contrast, IFFO1-M was detected at very low levels in only two of the eight control samples (Fig. 5A). Ten of the patient sera had more IFFO1-M DNA methylation than any of the positive control samples. The Receiver Operating Characteristics (ROC) analysis with an estimated area under the curve of 0.95 [95% CI, 0.8359 to 1] indicated a good discriminatory potential for the IFFO1-M marker (Fig. 5B). Since these results might have been influenced by differences in the way the control and case samples were collected and processed we continued the testing of IFFO1-M in serially collected serum samples from ovarian cancer patients.

### Verification of the IFFO1-M marker in a longitudinal screen for recurring ovarian cancer

We tested whether IFFO1-M marker could measure disease burden in serially collected serum samples of ovarian cancer patients and compared its performance to that of CA-125. This within-subject comparison eliminates the need of control samples since each patient serves as her own control. Serum samples were collected around the time of surgery (baseline samples) and during subsequent follow-up examinations for 16 ovarian cancer patients. The baseline samples from each of these 16 patients were used in the previous analysis (Table S3). Eleven of the baseline samples were collected before the surgery (mean = 11+/−5 days), while five of them were collected after the surgery (mean = 13+/−10 days) (Table S4). On average, 15 serum samples (ranging from 8 to 23) were collected at follow-up visits from each of the patients, with an average follow up time of 92 weeks (ranging from 37 to 246 weeks). All but three patients had abnormal CA-125 levels (≥35 U/ml) in the baseline samples.

**Figure 6:**
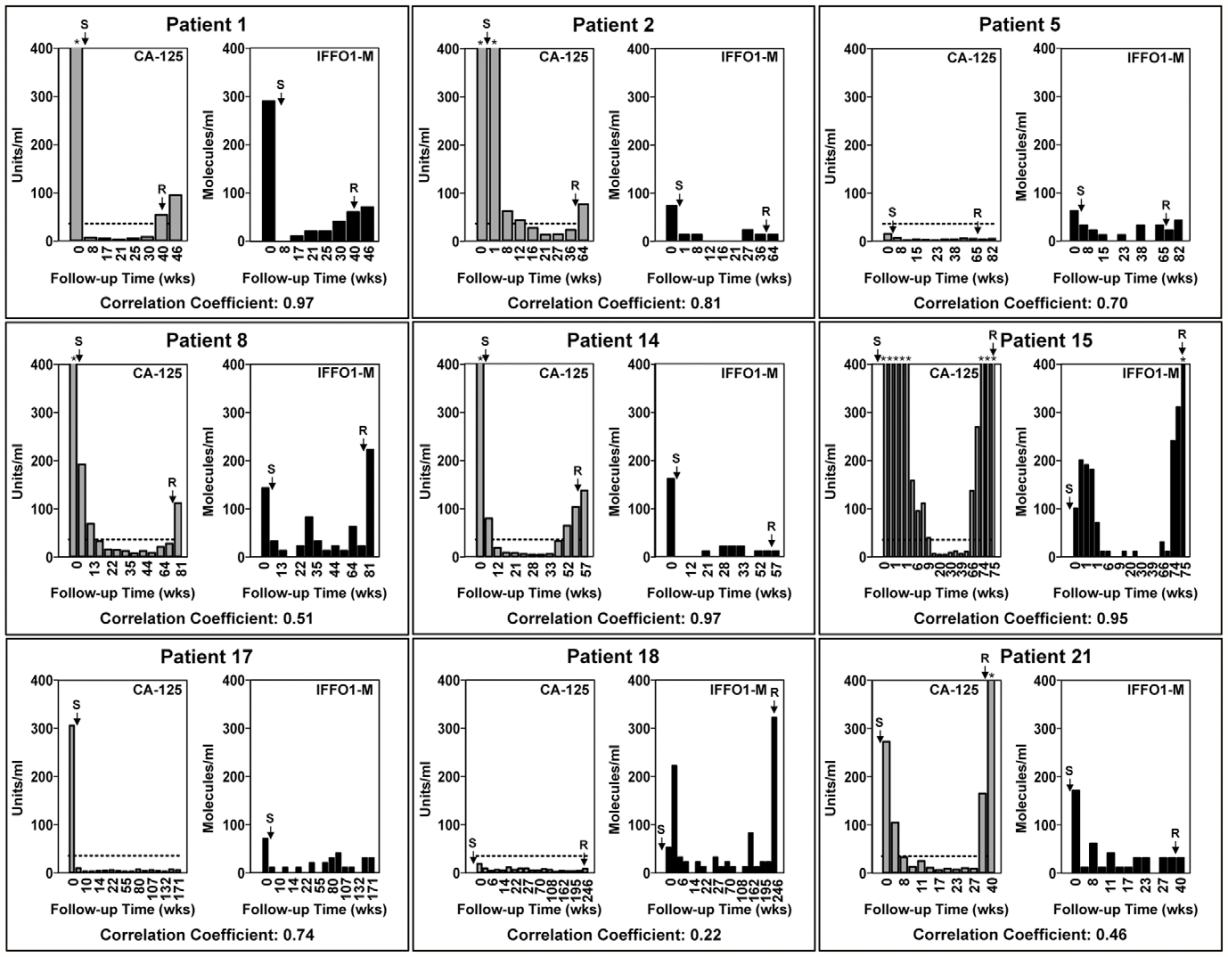
Comparison between the CA-125 and IFFO1-M performance in serially collected serum samples of nine ovarian cancer patients. Blood collected from ovarian cancer patients at the time of surgery (baseline samples) and at subsequent follow-up visits was used to measure CA-125 and IFFO1-M levels. The CA-125 levels (gray bars) are expressed in units/ml of blood, and the IFFO1-M methylation levels (black bars) are expresses as number of detected molecules/ml of sera on the Y-axis. The methylation analysis was performed using Digital MethyLight in DNA extracted from 100 µl of serum. The number of weeks since the baseline sample was collected is represented on the X-axis. The horizontal dashed line set at 35 u/ml represents the normal cut off value for CA-125. All patients except patients #5 and #18 had elevated levels of CA-125 in the baseline samples (>35 u/ml). The arrow labeled S indicates the time of surgery and the arrow labeled R indicates the time of tumor relapse as determined by CA-125 and/or imaging techniques. Due to the large range of CA-125 values we restricted the Y-axis to a scale of 400 for both of the markers, and we indicated the measurements that exceeded this scale by an asterisk. The values for these determinations are listed in the Table S4.

We used nine of the 16 patients with high levels of IFFO1-M in the baseline samples (Fig. 5A) to longitudinally compare the performance of the IFFO1-M marker to that of CA-125 in the serum samples collected during follow-up (Fig. 6). Of these, eight patients had baseline IFFO1-M serum levels well above background levels (Fig. 5A and Table S4). We included one additional patient (patient #18) with borderline baseline IFFO1-M levels, but with negative CA-125 measurements.

Two of the nine patients had normal CA-125 levels in the baseline samples (patients #5 and #18). Clinical recurrence occurred in all but one of the patients (patient #17). In the weeks immediately following surgery, both CA-125 levels and IFFO1-M DNA methylation measurements dropped in all patient samples relative to baseline. The decrease in both these markers paralleled the reduction in the tumor burden following the surgery. No increase of either CA-125 or IFFO1-M occurred after the initial drop in the patient #17 who never had a clinical relapse.

CA-125 levels eventually rose and exceeded normal levels (35 U/ml) in the follow-up samples of six of the eight patients with recurrent disease (patients #1, #2, #8, #14, #15, and #21). The IFFO1-M marker increased in four of the eight patients with recurrent disease. In three of these patients (patients #1, #8, and #15) the increase in IFFO1-M paralleled CA-125, whereas in one (patient #18) the IFFO1-M increase was not accompanied by an increase in the CA-125 levels. The increase of the IFFO-M occurred in three patients with serous ovarian cancer and in one with mucinous ovarian cancer. In combination, CA-125 and IFFO1-M DNA methylation markers tracked the disease status in eight of the nine analyzed patients. The IFFO1-M DNA methylation and CA-125 levels were correlated with each other in six out of nine patients (p<0.05, Pearson product-moment correlation test). The correlation coefficients were, 0.97, 0.81, 0.70, 0.97, 0.95, and 0.74 for patients #1, #2, #5, #14, #15 and #17 respectively. These data strongly suggest that the IFFO1-M marker correlates with disease status and that it may complement CA-125 in detecting disease recurrence in some cases.

## Discussion

We used a new strategy to identify blood-based candidate DNA methylation markers of ovarian cancer and to verify their potential to detect recurrent disease. We sought to circumvent some of the limitations associated with the biomarker development process [29]. To our knowledge this is the most extensive marker discovery study to date for ovarian cancer since most of the previous studies have relied on the use of candidate markers or limited screens.

One problem with biomarkers identified by high throughput technologies is their lack of sufficient specificity [29]. The use of a genome-scale screening approach presented us with the challenge of defining a clear marker selection strategy that would emphasize both marker sensitivity and specificity and help us prioritize among the hundreds of potential biomarkers. In most studies of epigenetic biomarkers for blood-based detection of cancer, marker specificity is initially inferred from normal vs. tumor tissue comparisons. We emphasized specificity of blood-based detection by directly comparing tumors from ovarian cancer patients to blood DNA from women without ovarian cancer, and eliminating markers found to be methylated in blood from age-matched healthy controls. We included ovarian cancer samples from four different ovarian cancer subtypes in the analysis (Table S1) to maximize marker sensitivity for detection for all of these types of ovarian cancer. During the verification process we counter-screened our markers with large quantities of PBL DNA and then with both serum- and plasma-derived DNA to exclude markers with low specificity.

DNA methylation markers generally suffer from poor clinical sensitivity [12]. In order to emphasize sensitivity in our marker selection strategy we used very stringent criteria that required consistently higher DNA methylation in all tumors than in any of the normal blood samples. We anticipated that this approach would enrich for markers with a high prevalence of DNA methylation in ovarian cancers, which in turn, would translate into a higher sensitivity for detection of ovarian cancer in patient blood than for markers with a lower frequency of tumor DNA methylation.

Promising biomarkers emerging from large-scale discovery efforts have often performed poorly when tested on independent validation samples [30]–[34] due in part to lack of randomization of case and control blood samples at baseline in observational diagnostic studies [34]. Diagnostic biomarker studies usually rely on subject selection on the basis of diagnosis, which can result in baseline differences between cases and controls [34]. This can lead to false-positive identification of disease-associated markers. Population-based cohort studies with prediagnostic blood samples can also be an excellent source for nested case-control comparisons (also referred to as a prospective-specimen collection, retrospective-blinded-evaluation (PRoBE) design [33]. However, the number of incident cases in cohorts is usually limiting and the high demand for these precious samples generally precludes their use for early-stage candidate DNA methylation biomarker evaluation. Also, since ovarian cancer is a disease with low incidence, the use of a prospective population-based study for early-phase ovarian cancer markers validation is not very practical.

In this study we tested an alternative verification scheme that evaluates the marker's correlation with a validated marker, CA-125, known to be associated with disease status in post-resection serially collected blood samples. This within-subject approach circumvents some of the drawbacks of traditional case-control designs, since each case serves as her own genetically matched control. The information regarding the ability of our top candidate marker, IFFO1-M, to measure disease status was extracted from the temporal pattern across many serial samples for each subject (8–21 samples per patient). The comparison to the validated biomarker CA-125 lent further support to the conclusion that IFFO1-M is measuring disease status.

The rapid decrease in the serum levels of IFFO1-M in all nine patients in the weeks immediately following surgery provides compelling evidence that IFFO1-M serum levels reflect tumor burden. In many cases, IFFO1-M closely tracked CA-125 serum levels in the post-resection serum specimens. IFFO1-M rose at the time of disease recurrence in three of the nine patients in a similar manner to CA-125, and even outperformed CA-125 in one additional patient in which CA-125 never increased over its normal values, despite disease relapse (Figure 6, Patient #18).

CA-125 surpassed IFFO1-M performance in three of the patients (#2, #14, and #21). This however, could be a direct consequence of the small volume of serum (100 µl) used for the DNA methylation analyses, and better performance of IFFO1-M should be expected in future studies using larger volumes of sera. In combination, CA-125 and IFFO1-M corresponded to relapse in seven of the eight patients with recurrent disease, suggesting that IFFO1-M may complement CA-125 in monitoring residual disease.

Despite increased efforts to identify new protein-based ovarian cancer biomarkers, CA-125 still remains the best marker for detecting early disease, up to three years in advance of the clinical diagnosis in some patients [35] and to monitor disease recurrence. This is the first time that a DNA methylation marker has been shown to have a concordant behavior with a protein marker with recognized clinical use. The analysis of post-resection serially collected samples may provide an effective method to evaluate whether a candidate marker has the potential to detect recurrent disease prior to the onset of symptoms or clinical evidence of disease and to help in the triage process of candidate markers that could be advanced for further analysis in valuable samples from larger population-based studies.

In conclusion, we have described here the potential of a new strategy to discover and verify candidate DNA methylation markers for detection of ovarian cancer, and we characterized a new marker, IFFO1-M, that can help enhance the performance of CA-125 in monitoring disease status. We expect this marker and any additional candidate markers emerging from this discovery pipeline to have a great chance of success in future validation stages of the marker development process.

## Supporting Information

Table S1 **Samples: source, histology, and alternative IDs in the various utilized assays.**
(DOC)

Table S2 **MethyLight primers and probes sequences.**
(DOC)

Table S3 **Clinical and pathological characteristics of the ovarian cancer patients and the age of the normal controls used for testing of the IFFO1-M in serum samples by digital MethyLight.**
(DOC)

Table S4 **CA-125 and IFFO1-M levels in the serially collected serum samples of nine patients with ovarian cancer.**
(DOC)
