# Supplementary material for: Genome-Scale Screen for DNA Methylation-Based Detection Markers for Ovarian Cancer
Source: PLoS One. 2011 Dec 7;6(12):e28141. doi: 10.1371/journal.pone.0028141 (PMC3233546; doi:10.1371/journal.pone.0028141)
Supplement: Table S1 — Samples: source, histology, and alternative IDs in the various utilized assays. (DOC) [file pone.0028141.s001.doc]

**Table S1.** Samples: source, histology, and alternative IDs in the various utilized assays

| Laird ID. | Source | Sample type | Histology | Illumina Infinium Assay | MethyLight | Digital MethyLight |
| --- | --- | --- | --- | --- | --- | --- |
| 14401 | Duke | tumor | Serous | 1 |  |  |
| 14402 | Duke | tumor | Serous | 2 |  |  |
| 14403 | Duke | tumor | Serous | 3 |  |  |
| 14404 | Duke | tumor | Serous | 4 |  |  |
| 14405 | Duke | tumor | Serous | 5 |  |  |
| 14406 | Duke | tumor | Serous | 6 |  |  |
| 14408 | Duke | tumor | Serous | 7 |  |  |
| 14409 | Duke | tumor | Serous | 8 |  |  |
| 14410 | Duke | tumor | Serous | 9 |  |  |
| 14411 | Duke | tumor | Serous | 10 |  |  |
| 14412 | Duke | tumor | Serous | 11 |  |  |
| 14414 | Duke | tumor | Serous | 12 |  |  |
| 14415 | Duke | tumor | Serous | 13 |  |  |
| 14416 | Duke | tumor | Serous | 14 |  |  |
| 14417 | Duke | tumor | Serous | 15 |  |  |
| 14418 | Duke | tumor | Serous | 16 |  |  |
| 14419 | Duke | tumor | Serous | 17 |  |  |
| 14420 | Duke | tumor | Serous | 18 |  |  |
| 14421 | Duke | tumor | Serous | 19 |  |  |
| 14422 | Duke | tumor | Serous | 20 |  |  |
| 14423 | Duke | tumor | Serous | 21 |  |  |
| 14424 | Duke | tumor | Serous | 22 |  |  |
| 14425 | Duke | tumor | Serous | 23 |  |  |
| 14426 | Duke | tumor | Serous | 24 |  |  |
| 14428 | Duke | tumor | Serous | 25 |  |  |
| 14429 | Duke | tumor | Serous | 26 |  |  |
| 14430 | Duke | tumor | Serous | 27 |  |  |
| 12762 | USC | tumor | Serous | 28 | 14 |  |
| **Supplemental Table 1**. Sample’s source, histology, and alternative IDs in the various utilized assays (continued) | | | | | | |
| Laird ID. | Source | Sample type | Histology | Illumina Infinium Assay ID | MethyLight ID | Digital MethyLight ID |
| 12764 | USC | tumor | Serous | 29 | 15 |  |
| 12854 | USC | tumor | Endometrioid | 30 | 7 |  |
| 12867 | USC | tumor | Endometrioid | 31 | 8 |  |
| 12853 | USC | tumor | Endometrioid | 32 | 6 |  |
| 12862 | USC | tumor | Endometrioid | 33 | 5 |  |
| 12864 | USC | tumor | Mixed Clear Cell/Endometrioid | 34 | 4 |  |
| 12865 | USC | tumor | Clear Cell | 35 | 2 |  |
| 12767 | USC | tumor | Clear Cell | 36 | 1 |  |
| 12855 | USC | tumor | Clear Cell | 37 | 3 |  |
| 12766 | USC | tumor | Mucinous | 38 | 9 |  |
| 12852 | USC | tumor | Mucinous | 39 | 10 |  |
| 12863 | USC | tumor | Mucinous | 40 | 12 |  |
| 12866 | USC | tumor | Mucinous | 41 | 11 |  |
| 12868 | USC | tumor | Serous |  | 13 |  |
| 10703 | HemaCare | Control PBL |  | 1 |  |  |
| 10704 | HemaCare | Control PBL |  | 2 |  |  |
| 10707 | HemaCare | Control PBL |  | 3 |  |  |
| 10705 | HemaCare | Control PBL |  | 4 |  |  |
| 10706 | HemaCare | Control PBL |  | 5 |  |  |
| 10708* | HemaCare | Control PBL |  | 6 |  |  |
| 10709 | HemaCare | Control PBL |  | 7 |  |  |
| 10710* | HemaCare | Control PBL |  | 8 |  |  |
| 10711 | HemaCare | Control PBL |  | 9 |  |  |
| 10712 | HemaCare | Control PBL |  | 10 |  |  |
| 11162 | HemaCare | Control plasma |  |  | 1 |  |
| 11163 | HemaCare | Control plasma |  |  | 2 |  |
| 11164 | HemaCare | Control plasma |  |  | 3 |  |
| 11165 | HemaCare | Control plasma |  |  | 4 |  |
| 11166 | HemaCare | Control plasma |  |  | 5 |  |
| 11167 | HemaCare | Control plasma |  |  | 6 |  |
| **Supplemental Table 1**. Sample’s source, histology, and alternative IDs in the various utilized assays (continued) | | | | | | |
| Laird ID. | Source | Sample type | Histology | Illumina Infinium Assay ID | MethyLight ID | Digital MethyLight ID |
| 11168 | HemaCare | Control plasma |  |  | 7 |  |
| 11169 | HemaCare | Control plasma |  |  | 8 |  |
| 11170 | HemaCare | Control plasma |  |  | 9 |  |
| 11171 | HemaCare | Control plasma |  |  | 10 |  |
| 11480 | Innovative Res | Control sera |  |  |  | 1 |
| 11481 | Innovative Res | Control sera |  |  |  | 2 |
| 11482 | Innovative Res | Control sera |  |  |  | 3 |
| 11681 | Innovative Res | Control sera |  |  |  | 4 |
| 11682 | Innovative Res | Control sera |  |  |  | 5 |
| 11683 | Innovative Res | Control sera |  |  |  | 6 |
| 11684 | Innovative Res | Control sera |  |  |  | 7 |
| 11973 | Innovative Res | Control sera |  |  |  | 8 |
| 10817 | Innsbruck | Patient sera | Serous |  |  | 1 |
| 10818 | Innsbruck | Patient sera | Serous |  |  | 2 |
| 10820 | Innsbruck | Patient sera | Mucinous |  |  | 4 |
| 10821 | Innsbruck | Patient sera | Serous |  |  | 5 |
| 10822 | Innsbruck | Patient sera | Serous |  |  | 6 |
| 10824 | Innsbruck | Patient sera | Mucinous |  |  | 8 |
| 10825 | Innsbruck | Patient sera | Serous |  |  | 9 |
| 10828 | Innsbruck | Patient sera | Endometrioid |  |  | 12 |
| 10829 | Innsbruck | Patient sera | Serous |  |  | 13 |
| 10830 | Innsbruck | Patient sera | Endometrioid |  |  | 14 |
| 10831 | Innsbruck | Patient sera | Serous |  |  | 15 |
| 10832 | Innsbruck | Patient sera | Serous |  |  | 16 |
| 10833 | Innsbruck | Patient sera | Endometrioid |  |  | 17 |
| 10834 | Innsbruck | Patient sera | Serous |  |  | 18 |
| 10835 | Innsbruck | Patient sera | Serous |  |  | 19 |
| 10837 | Innsbruck | Patient sera | Serous |  |  | 21 |
