# Supplementary material for: Genome-Scale Screen for DNA Methylation-Based Detection Markers for Ovarian Cancer
Source: PLoS One. 2011 Dec 7;6(12):e28141. doi: 10.1371/journal.pone.0028141 (PMC3233546; doi:10.1371/journal.pone.0028141)
Supplement: Table S2 — MethyLight primers and probes sequences. (DOC) [file pone.0028141.s002.doc]

| **Table S2.** MethyLight Primers and Probes Sequences | | | | | | |
| --- | --- | --- | --- | --- | --- | --- |
| **HUGO Nomen**  **clature** | **Chromosomal**  **Location** | **Amplicon Location**  **UCSC**  **(Feb 2009)** | **Reaction ID** | **Forward Primer** | **Reverse Primer** | **Probe** |
| ***CD6*** | 11 | 60739218-60739303 | HB-772 | GAATAGTAAAGGGTAGAGTAGATTTGCG | CCTATAAAAAACACCATCTTAAAACCG | 6-FAM-TAAACACGACCGTTATACG-MGBNFQ |
| **OSM** | 22 | 30661875-30661967 | HB-765 | GGGCGGGTATAGTTATCGTTTAT | TACCTTCCGAACCATTACGC | 6FAM-CGACCACGTAACTACCGATACCCCGTTA-BHQ1 |
| ***TMEM149*** | 19 | 36233422-36233530 | HB-820 | AGTAAGTTGATGTCGAAGTGAAATACG | TCCTTCCCAAACTCCCGA | 6FAM-TTCAACATAAAAACCTTCTTCTACCGCCCC-BHQ1 |
| ***PTPN7*** | 1 | 202129775-202129885 | HB-807 | TCGTTTTTTTTTGTTGTTATTTACGTA | AACCCCAAAAAAACACAAACG | 6FAM-ACAACCGACCCCTAATAACAACGACAACA-BHQ1 |
| ***TMC8*** | 17 | 76127469-76127564 | HB-819 | GTAGAGCGGTAGATTCGGGTAA | TAAACTAAAAACGAACCCTTCCG | 6FAM-CCCCTCTACGCCGACGTCGAA-BHQ1 |
| ***IFFO1*** | 12 | 6665179-6665271 | HB-757 | TCGAATAACGGATTTATGGTTGC | GCCGCATTAACTCTTCTAACTCG | 6 FAM-CCCTACTCCTACACCGATCTACATCTCCCAA-BHQ1 |
| ***BIN2*** | 12 | 51718059-51718168 | HB-822 | TGGAGTAGGTCGGTTCGGAG | ATTCAATAAAACCGAAATTTCGAA | 6FAM-CGAAAACTAACCTCGAACGAACGCTACCTA-BHQ1 |
| ***C160rf54*** | 16 | 29757309-29757378 | HB-796 | GGTGTAGGGCGGTTCGTT | AACGATACCCTCCCTTCCG | 6FAM-CTTCATCCGAAACCGAACCTCTACGTCTAA-BHQ1 |
| ***TBC1D10C*** | 11 | 67171693-67171804 | HB-802 | AGGATTTGGTGTAGTTTTTCGAGT | CCAATAAATCCATAACGATCGACCTAACG | 6FAM-AACCAAACCCGCTAAACTCTAAATCGAACC-BHQ1 |
| ***TNFRSF25*** | 1 | 6526161-6526229 | HB-080 | GCGGAATTACGACGGGTAGA | ACTCCATAACCCTCCGACGA | 6FAM-CGCCCAAAAACTTCCCGACTCCGTA-BHQ-1 |
| ***PSD4*** | 2 | 113931499-113931588 | HB-751 | GTGTTGTGGTGAGGAGGAGC | AAAACACTTCCTAACTCACAATCG | 6FAM-TATAACTATAACGACTAAAACTTCCGACTT-MGBNFQ |

The coordinates for the MethyLight amplicons are given using the February 2009 Assembly from the University of California Santa Cruz (UCSC) Genome Browser. The primers and probes sequences are written in the 5’ to 3’ orientation. All probes contain at the 5’ end a 6FAM fluorophore and either a Black Hole Quencher (BHQ) or a Minor Groove Binding Non-Fluorescent Quencher (MGBNFQ) and the 3’ end
