# Supplementary material for: Genome-Scale Screen for DNA Methylation-Based Detection Markers for Ovarian Cancer
Source: PLoS One. 2011 Dec 7;6(12):e28141. doi: 10.1371/journal.pone.0028141 (PMC3233546; doi:10.1371/journal.pone.0028141)
Supplement: Table S3 — Clinical and pathological characteristics of the ovarian cancer patients and the age of the normal controls used for testing of the IFFO1-M in serum samples by digital MethyLight. (DOC) [file pone.0028141.s003.doc]

| **Table S3**. Clinical and Pathological Characteristics of the Ovarian Cancer Patients and the Age of the Normal Controls used for testing of the IFFO1-M in Serum Samples by Digital MethyLight | | | | | | | | | | | | |
| --- | --- | --- | --- | --- | --- | --- | --- | --- | --- | --- | --- | --- |
| **Laird ID** | **Case/Control ID** | **Sample**  **Type** | **Patient**  **Age** | **Date of Surgery** | **Histology** | **FIGO** | **Grade** | **Weeks of Follow- up** | **Date of Relapse** | **Date of Death** | **Baseline Sample**  **Status** | **Tested**  **for**  **recurrence** |
| 10838 | 1 | Case | 63 | 10/09/95 | Serous | 3C | 3 | 46 | 7/18/00 | 8/12/01 | Preoperative | yes |
| 10846 | 2 | Case | 58 | 10/17/97 | Serous | 3C | 2 | 64 | 12/4/02 | 9/15/04 | Preoperative | yes |
| 10875 | 4 | Case | 48 | 3/20/96 | Mucinous | 3C | 3 | 104 | 3/10/02 | 7/14/03 | Preoperative | yes |
| 10898 | 5 | Case | 73 | 2/8/00 | Serous | 3B | 3 | 82 | 7/27/05 | 6/3/07 | Preoperative | yes |
| 10910 | 6 | Case | 61 | 4/20/98 | Serous | 3C | 2 | 61 | 6/2/03 | 3/11/04 | Preoperative | yes |
| 10952 | 8 | Case | NA | 5/26/94 | Mucinous | 3C | NA | 81 | 11/17/99 | 11/19/00 | Preoperative | yes |
| 10965 | 9 | Case | 63 | 1/29/97 | Serous | 3C | 3 | 127 | 6/25/03 | 8/10/07 | Preoperative | yes |
| 11019 | 12 | Case | 58 | 3/19/98 | Endometrioid | 3C | 3 | 54 | 3/12/03 | 9/23/03 | Preoperative | yes |
| 11030 | 13 | Case | 67 | 6/27/95 | Serous | 3C | 3 | 84 | 1/30/01 | 7/27/02 | Postoperative | yes |
| 11047 | 14 | Case | 56 | 4/5/94 | Endometrioid | 3C | 3 | 57 | 5/5/99 | 12/12/99 | Preoperative | yes |
| 11060 | 15 | Case | 48 | 1/30/92 | Serous | 4 | 2 | 75 | 7/19/97 | 12/9/97 | Postoperative | yes |
| 11081 | 16 | Case | 63 | 5/6/98 | Serous | 3C | 2 | 37 | 12/8/02 | 9/16/03 | Preoperative | yes |
| 11089 | 17 | Case | 64 | 9/10/97 | Endometrioid | 3C | 3 | 171 |  | 9/29/12 | Preoperative | yes |
| 11107 | 18 | Case | 60 | 2/25/92 | Serous | 3A | 3 | 246 | 11/16/00 | 11/22/06 | Postoperative | yes |
| 11127 | 19 | Case | 58 | 8/19/97 | Serous | 3C | 2 | 95 | 7/16/03 | 7/1/07 | Postoperative | yes |
| 11149 | 21 | Case | 59 | 10/25/99 | Serous | 3C | 2 | 40 | 7/22/04 | 1/27/05 | Postoperative | yes |
| 10875 | 4 | Case | 48 | 3/20/96 | Mucinous | 3C | 3 | 104 | 3/9/98 | 7/13/99 | Preoperative | no |
| 10910 | 6 | Case | 61 | 4/20/98 | Serous | 3C | 2 | 61 | 6/1/99 | 3/10/01 | Preoperative | no |
| 10965 | 9 | Case | 63 | 1/29/97 | Serous | 3C | 3 | 127 | 6/24/99 | 8/9/03 | Preoperative | no |
| 11019 | 12 | Case | 58 | 3/19/98 | Endometrioid | 3C | 3 | 54 | 3/11/99 | 9/22/99 | Preoperative | no |
| 11030 | 13 | Case | 67 | 6/27/95 | Serous | 3C | 3 | 84 | 1/29/97 | 7/26/98 | Postoperative | no |
| 11081 | 16 | Case | 63 | 05/6/98 | Serous | 3C | 2 | 37 | 12/7/98 | 9/15/99 | Preoperative | no |
| 11127 | 19 | Case | 58 | 8/19/97 | Serous | 3C | 2 | 95 | 07/15/99 | 6/30/03 | Postoperative | no |
| 11480 | 1 | Control | 57 |  |  |  |  |  |  |  |  |  |
| 11481 | 2 | Control | 56 |  |  |  |  |  |  |  |  |  |
| 11482 | 3 | Control | 57 |  |  |  |  |  |  |  |  |  |
| 11681 | 4 | Control | 50 |  |  |  |  |  |  |  |  |  |
| 11682 | 5 | Control | 51 |  |  |  |  |  |  |  |  |  |
| 11683 | 6 | Control | 52 |  |  |  |  |  |  |  |  |  |
| 11684 | 7 | Control | 62 |  |  |  |  |  |  |  |  |  |
| 11973 | 8 | Control | NA |  |  |  |  |  |  |  |  |  |

NA = not available
