# Supplementary material for: Genome-Scale Screen for DNA Methylation-Based Detection Markers for Ovarian Cancer
Source: PLoS One. 2011 Dec 7;6(12):e28141. doi: 10.1371/journal.pone.0028141 (PMC3233546; doi:10.1371/journal.pone.0028141)
Supplement: Table S4 — CA-125 and IFFO1-M levels in the serially collected serum samples of nine patients with ovarian cancer. (DOC) [file pone.0028141.s004.doc]

**Table S4**. CA-125 and IFFO1-M levels in the serially collected serum samples of nine patients with ovarian cancer

| **Patient 1** | | | | | | |
| --- | --- | --- | --- | --- | --- | --- |
| **Sample ID** | **Collection Date** | **Surgery Date** | **Baseline Sample** | **Weeks of Follow-up** | **CA-125* Levels (U/ml)** | **IFFO1-M**  **No. of Methylated Molec/100µl** |
| 1_1 | 9/27/95 | 10/9/95 | Preoperative | 0 | 3570 | 29 |
| 1_2 | 11/24/95 |  |  | 8 | 6 | 0 |
| 1_3 | 1/25/96 |  |  | 17 | 4 | 1 |
| 1_4 | 2/23/96 |  |  | 21 | 2 | 2 |
| 1_5 | 3/22/96 |  |  | 25 | 4 | 2 |
| 1_6 | 4/26/96 |  |  | 30 | 7 | 4 |
| 1_7 | 7/3/96 |  |  | 40 | 53 | 6 |
| 1_8 | 8/14/96 |  |  | 46 | 94 | 7 |

| **Patient 2** | | | | | | |
| --- | --- | --- | --- | --- | --- | --- |
| **Sample ID** | **Collection Date** | **Surgery Date** | **Baseline Sample** | **Weeks of Follow-up** | **CA-125* Levels (U/ml)** | **IFFO1-M**  **No. of Methylated Molec/100µl** |
| 2_1 | 10/8/97 | 10/17/97 | Preoperative | 0 | 826 | 7 |
| 2_2 | 10/13/97 |  |  | 1 | 545 | 1 |
| 2_3 | 12/2/97 |  |  | 8 | 61 | 1 |
| 2_4 | 12/30/97 |  |  | 12 | 42 | 0 |
| 2_5 | 1/28/98 |  |  | 16 | 26 | 0 |
| 2_6 | 3/4/98 |  |  | 21 | 12 | 0 |
| 2_7 | 4/16/98 |  |  | 27 | 13 | 2 |
| 2_8 | 6/15/98 |  |  | 36 | 22 | 1 |
| 2_9 | 9/21/98 |  |  | 50 | 82 | NA |
| 2_10 | 12/28/98 |  |  | 64 | 75 | 1 |

**Supplemental Table 4 (continued)**

| **Patient 4** | | | | | | |
| --- | --- | --- | --- | --- | --- | --- |
| **Sample ID** | **Collection Date** | **Surgery Date** | **Baseline Sample** | **Weeks of Follow-up** | **CA-125* Levels (U/ml)** | **IFFO1-M**  **No. of Methylated Molec/100µl** |
| 4_1 | 3/12/1996 | 3/20/96 | Preoperative | 0 | 553 | 3 |
| 4_2 | 5/3/1996 |  |  | 7 | 20 |  |
| 4_3 | 5/29/1996 |  |  | 11 | 14 |  |
| 4_4 | 6/28/1996 |  |  | 15 | 11 |  |
| 4_5 | 8/7/1996 |  |  | 21 | 7 |  |
| 4_6 | 9/4/1996 |  |  | 25 | 7 |  |
| 4_7 | 10/1/1996 |  |  | 29 | 4 |  |
| 4_8 | 11/28/1996 |  |  | 37 | 6 |  |
| 4_9 | 12/19/1996 |  |  | 40 | 4 |  |
| 4_10 | 1/17/1997 |  |  | 44 | 3 |  |
| 4_11 | 2/7/1997 |  |  | 47 | 2 |  |
| 4_12 | 2/8/1997 |  |  | 48 | 2 |  |
| 4_13 | 2/28/1997 |  |  | 50 | 3 |  |
| 4_14 | 3/21/1997 |  |  | 53 | 2 |  |
| 4_15 | 4/11/1997 |  |  | 56 | 2 |  |
| 4_16 | 5/6/1997 |  |  | 60 | 2 |  |
| 4_17 | 5/27/1997 |  |  | 63 | 3 |  |
| 4_18 | 7/1/1997 |  |  | 68 | 2 |  |
| 4_19 | 8/21/1997 |  |  | 75 | 4 |  |
| 4_20 | 11/19/1997 |  |  | 88 | 13 |  |
| 4_21 | 2/18/1998 |  |  | 101 | 246 |  |
| 4_22 | 3/4/1998 |  |  | 103 | 267 |  |
| 4_23 | 3/10/1998 |  |  | 104 | 286 |  |

| **Patient 5** | | | | | | |
| --- | --- | --- | --- | --- | --- | --- |
| **Sample ID** | **Collection Date** | **Surgery Date** | **Baseline Sample** | **Weeks of Follow-up** | **CA-125* Levels (U/ml)** | **IFFO1-M**  **No. of Methylated Molec/100µl** |
| 5_1 | 1/18/00 | 2/8/00 | Preoperative | 0 | 14 | 6 |
| 5_2 | 3/13/00 |  |  | 8 | 6 | 3 |
| 5_3 | 4/5/00 |  |  | 11 | 1 | 2 |
| 5_4 | 5/4/00 |  |  | 15 | 3 | 1 |
| 5_5 | 6/2/00 |  |  | 19 | 2 | 0 |
| 5_6 | 6/29/00 |  |  | 23 | 1 | 1 |
| 5_7 | 7/26/00 |  |  | 27 | 3 | 0 |
| 5-8 | 10/12/00 |  |  | 38 | 3 | 3 |
| 5_9 | 1/11/01 |  |  | 51 | 5 | 0 |
| 5_10 | 4/18/01 |  |  | 65 | 4 | 3 |
| 5_11 | 7/25/01 |  |  | 79 | 3 | 2 |
| 5_12 | 8/15/01 |  |  | 82 | 4 | 4 |

**Supplemental Table 4 (continued)**

| **Patient 6** | | | | | | |
| --- | --- | --- | --- | --- | --- | --- |
| **Sample ID** | **Collection Date** | **Surgery Date** | **Baseline Sample** | **Weeks of Follow-up** | **CA-125* Levels (U/ml)** | **IFFO1-M**  **No. of Methylated Molec/100µl** |
| 6_1 | 4/7/1998 | 4/20/98 | Preoperative | 0 | 7410 | 2 |
| 6_2 | 5/26/1998 |  |  | 7 | 2160 |  |
| 6_3 | 6/17/1998 |  |  | 10 | 2003 |  |
| 6_4 | 7/9/1998 |  |  | 13 | 1374 |  |
| 6_5 | 8/6/1998 |  |  | 17 | 449 |  |
| 6_6 | 9/21/1998 |  |  | 24 | 125 |  |
| 6_7 | 10/20/1998 |  |  | 28 | 58 |  |
| 6-8 | 11/3/1998 |  |  | 30 | 37 |  |
| 6_9 | 11/18/1998 |  |  | 32 | 35 |  |
| 6_10 | 12/21/1998 |  |  | 37 | 23 |  |
| 6_11 | 3/31/1999 |  |  | 51 | 17 |  |
| 6_12 | 4/20/1999 |  |  | 54 | 89 |  |
| 6_13 | 6/10/1999 |  |  | 61 | 3190 |  |

| **Patient 8** | | | | | | |
| --- | --- | --- | --- | --- | --- | --- |
| **Sample ID** | **Collection Date** | **Surgery Date** | **Baseline Sample** | **Weeks of Follow-up** | **CA-125* Levels (U/ml)** | **IFFO1-M**  **No. of Methylated Molec/100µl** |
| 8_1 | 5/9/94 | 5/26/94 | Preoperative | 0 | 561 | 14 |
| 8_2 | 7/11/94 |  |  | 9 | 191 | 3 |
| 8_3 | 8/10/94 |  |  | 13 | 68 | 1 |
| 8_4 | 9/9/94 |  |  | 18 | 32 | 0 |
| 8_5 | 10/7/94 |  |  | 22 | 15 | 2 |
| 8_6 | 11/11/94 |  |  | 27 | 14 | 8 |
| 8_7 | 1/6/95 |  |  | 35 | 12 | 3 |
| 8_8 | 2/16/95 |  |  | 40 | 7 | 1 |
| 8_9 | 3/16/95 |  |  | 44 | 12 | 2 |
| 8_10 | 5/3/95 |  |  | 51 | 8 | 1 |
| 8_11 | 8/1/95 |  |  | 64 | 20 | 6 |
| 8_12 | 8/18/95 |  |  | 67 | 27 | 2 |
| 8_13 | 11/29/95 |  |  | 81 | 111 | 22 |

**Supplemental Table 4 (continued)**

| **Patient 9** | | | | | | |
| --- | --- | --- | --- | --- | --- | --- |
| **Sample ID** | **Collection Date** | **Surgery Date** | **Baseline Sample** | **Weeks of Follow-up** | **CA-125* Levels (U/ml)** | **IFFO1-M**  **No. of Methylated Molec/100µl** |
| 9_1 | 1/15/1997 | 1/29/97 | Preoperative | 0 | 425 | 2 |
| 9_2 | 1/22/1997 |  |  | 1 | 463 |  |
| 9_3 | 3/18/1997 |  |  | 9 | 8 |  |
| 9_4 | 4/10/1997 |  |  | 12 | 5 |  |
| 9_5 | 5/7/1997 |  |  | 16 | 3 |  |
| 9_6 | 6/11/1997 |  |  | 21 | 3 |  |
| 9_7 | 6/16/1997 |  |  | 22 | 2 |  |
| 9_8 | 7/8/1997 |  |  | 25 | 3 |  |
| 9_9 | 8/5/1997 |  |  | 29 | NA |  |
| 9_10 | 8/19/1997 |  |  | 31 | 6 |  |
| 9_11 | 9/16/1997 |  |  | 35 | 3 |  |
| 9_12 | 10/8/1997 |  |  | 38 | 3 |  |
| 9_13 | 10/30/1997 |  |  | 41 | 4 |  |
| 9_14 | 12/9/1997 |  |  | 47 | 5 |  |
| 9_15 | 4/16/1998 |  |  | 65 | 4 |  |
| 9_16 | 7/29/1998 |  |  | 80 | 1 |  |
| 9_17 | 10/28/1998 |  |  | 93 | 3 |  |
| 9_18 | 3/23/1999 |  |  | 114 | 13 |  |
| 9_19 | 6/11/1999 |  |  | 125 | 46 |  |
| 9_20 | 6/25/1999 |  |  | 127 | 55 |  |

| **Patient 12** | | | | | | |
| --- | --- | --- | --- | --- | --- | --- |
| **Sample ID** | **Collection Date** | **Surgery Date** | **Baseline Sample** | **Weeks of Follow-up** | **CA-125* Levels (U/ml)** | **IFFO1-M**  **No. of Methylated Molec/100µl** |
| 12_1 | 3/9/1998 | 3/19/98 | Preoperative | 0 | 87 | 3 |
| 12_2 | 3/18/1998 |  |  | 1 | 112 |  |
| 12_3 | 4/3/1998 |  |  | 4 | 82 |  |
| 12_4 | 4/30/1998 |  |  | 7 | 21 |  |
| 12_5 | 5/26/1998 |  |  | 11 | 10 |  |
| 12_6 | 7/3/1998 |  |  | 17 | 13 |  |
| 12_7 | 7/24/1998 |  |  | 20 | 7 |  |
| 12_8 | 8/14/1998 |  |  | 23 | 10 |  |
| 12_9 | 12/1/1998 |  |  | 38 | 8 |  |
| 12_10 | 2/23/1999 |  |  | 50 | 227 |  |
| 12_11 | 3/24/1999 |  |  | 54 | 1000 |  |

**Supplemental Table 4 (continued)**

| **Patient 13** | | | | | | |
| --- | --- | --- | --- | --- | --- | --- |
| **Sample ID** | **Collection Date** | **Surgery Date** | **Baseline Sample** | **Weeks of Follow-up** | **CA-125* Levels (U/ml)** | **IFFO1-M**  **No. of Methylated Molec/100µl** |
| 13_1 | 7/14/1995 | 6/27/95 | Postoperative | 0 | 1215 | 3 |
| 13_2 | 8/31/1995 |  |  | 7 | 313 |  |
| 13_3 | 10/5/1995 |  |  | 12 | 99 |  |
| 13_4 | 11/8/1995 |  |  | 17 | 43 |  |
| 13_5 | 12/20/1995 |  |  | 23 | 18 |  |
| 13_6 | 1/23/1996 |  |  | 28 | 9 |  |
| 13_7 | 2/21/1996 |  |  | 32 | 7 |  |
| 13_8 | 3/7/1996 |  |  | 34 | 5 |  |
| 13_9 | 4/22/1996 |  |  | 40 | 12 |  |
| 13_10 | 5/29/1996 |  |  | 46 | 19 |  |
| 13_11 | 7/2/1996 |  |  | 51 | 28 |  |
| 13_12 | 8/9/1996 |  |  | 56 | 47 |  |
| 13_13 | 9/11/1996 |  |  | 61 | 50 |  |
| 13_14 | 10/9/1996 |  |  | 65 | 56 |  |
| 13_15 | 1/8/1997 |  |  | 78 | 279 |  |
| 13_16 | 2/12/1997 |  |  | 83 | 286 |  |
| 13_17 | 2/20/1997 |  |  | 84 | 348 |  |

| **Patient 14** | | | | | | |
| --- | --- | --- | --- | --- | --- | --- |
| **Sample ID** | **Collection Date** | **Surgery Date** | **Baseline Sample** | **Weeks of Follow-up** | **CA-125* Levels (U/ml)** | **IFFO1-M**  **No. of Methylated Molec/100µl** |
| 14_1 | 3/31/94 | 4/5/94 | Preoperative | 0 | 1095 | 16 |
| 14_2 | 5/18/94 |  |  | 7 | 79 | 0 |
| 14_3 | 6/22/94 |  |  | 12 | 18 | 0 |
| 14_4 | 7/18/94 |  |  | 16 | 8 | 0 |
| 14_5 | 8/22/94 |  |  | 21 | 7 | 1 |
| 14_6 | 9/20/94 |  |  | 25 | 5 | 0 |
| 14_7 | 10/11/94 |  |  | 28 | 4 | 2 |
| 14_8 | 10/18/94 |  |  | 29 | 4 | 2 |
| 14_9 | 11/17/94 |  |  | 33 | 5 | 2 |
| 14_10 | 2/23/95 |  |  | 47 | 32 | 0 |
| 14_11 | 3/30/95 |  |  | 52 | 64 | 1 |
| 14_12 | 4/25/95 |  |  | 56 | 103 | 1 |
| 14_13 | 5/4/95 |  |  | 57 | 137 | 1 |

**Supplemental Table 4** (continued)

| **Patient 15** | | | | | | |
| --- | --- | --- | --- | --- | --- | --- |
| **Sample ID** | **Collection Date** | **Surgery Date** | **Baseline Sample** | **Weeks of Follow-up** | **CA-125* Levels (U/ml)** | **IFFO1-M**  **No. of Methylated Molec/100µl** |
| 15_1 | 2/6/92 | 1/30/92 | Postoperative | 0 | 1240 | 10 |
| 15_2 | 2/9/92 |  |  | 0 | 1455 | 20 |
| 15_3 | 2/10/92 |  |  | 1 | 1265 | 19 |
| 15_4 | 2/11/92 |  |  | 1 | 1160 | 18 |
| 15_5 | 2/12/92 |  |  | 1 | 1270 | 7 |
| 15_6 | 3/10/92 |  |  | 5 | 158 | 1 |
| 15_7 | 3/17/92 |  |  | 6 | 95 | 1 |
| 15_8 | 3/24/92 |  |  | 7 | 111 | 0 |
| 15_9 | 4/10/92 |  |  | 9 | 39 | 0 |
| 15_10 | 5/20/92 |  |  | 15 | 6 | 1 |
| 15_11 | 6/25/92 |  |  | 20 | 5 | 0 |
| 15_12 | 7/28/92 |  |  | 25 | 5 | 1 |
| 15_13 | 9/1/92 |  |  | 30 | 9 | 0 |
| 15_14 | 9/30/92 |  |  | 34 | 12 | 0 |
| 15_15 | 11/5/92 |  |  | 39 | 6 | 0 |
| 15_16 | 2/5/93 |  |  | 52 | 11 | 0 |
| 15_17 | 5/11/93 |  |  | 66 | 137 | 3 |
| 15_18 | 5/25/93 |  |  | 68 | 269 | 1 |
| 15_19 | 7/7/93 |  |  | 74 | 3630 | 24 |
| 15_20 | 7/13/93 |  |  | 75 | 4810 | 31 |
| 15_21 | 7/17/93 |  |  | 75 | 5580 | 42 |

| **Patient 16** | | | | | | |
| --- | --- | --- | --- | --- | --- | --- |
| **Sample ID** | **Collection Date** | **Surgery Date** | **Baseline Sample** | **Weeks of Follow-up** | **CA-125* Levels (U/ml)** | **IFFO1-M**  **No. of Methylated Molec/100µl** |
| 16_1 | 4/27/1998 | 05/6/98 | Preoperative | 0 | 210 | 5 |
| 16_2 | 6/10/1998 |  |  | 6 | 28 |  |
| 16_3 | 7/1/1998 |  |  | 9 | 18 |  |
| 16_4 | 7/22/1998 |  |  | 12 | 14 |  |
| 16_5 | 8/14/1998 |  |  | 16 | 16 |  |
| 16_6 | 9/4/1998 |  |  | 19 | 16 |  |
| 16_7 | 11/24/1998 |  |  | 30 | 67 |  |
| 16_8 | 1/12/1999 |  |  | 37 | 77 |  |

**Supplemental Table 4 (continued)**

| **Patient 17** | | | | | | |
| --- | --- | --- | --- | --- | --- | --- |
| **Sample ID** | **Collection Date** | **Surgery Date** | **Baseline Sample** | **Weeks of Follow-up** | **CA-125* Levels (U/ml)** | **IFFO1-M**  **No. of Methylated Molec/100µl** |
| 17_1 | 9/3/97 | 9/10/97 | Preoperative | 0 | 305 | 7 |
| 17_2 | 10/17/97 |  |  | 6 | 9 | 1 |
| 17_3 | 11/12/97 |  |  | 10 | 2 | 0 |
| 17_4 | 12/11/97 |  |  | 14 | 2 | 1 |
| 17_5 | 1/13/98 |  |  | 19 | 3 | 0 |
| 17_6 | 2/6/98 |  |  | 22 | 4 | 1 |
| 17_7 | 3/3/98 |  |  | 26 | 5 | 0 |
| 17_8 | 3/27/98 |  |  | 29 | 3 | 2 |
| 17_9 | 6/24/98 |  |  | 42 | NA | 0 |
| 17_10 | 9/23/98 |  |  | 55 | 2 | 0 |
| 17_11 | 12/16/98 |  |  | 67 | 2 | 2 |
| 17_12 | 3/18/99 |  |  | 80 | 6 | 3 |
| 17_13 | 6/17/99 |  |  | 93 | 3 | 4 |
| 17_14 | 9/22/99 |  |  | 107 | 5 | 1 |
| 17_15 | 12/15/99 |  |  | 119 | 3 | 1 |
| 17_16 | 3/17/00 |  |  | 132 | 2 | 0 |
| 17_17 | 6/20/00 |  |  | 146 | 6 | 3 |
| 17_18 | 12/13/00 |  |  | 171 | 5 | 3 |

| **Patient 18** | | | | | | |
| --- | --- | --- | --- | --- | --- | --- |
| **Sample ID** | **Collection Date** | **Surgery Date** | **Baseline Sample** | **Weeks of Follow-up** | **CA-125* Levels (U/ml)** | **IFFO1-M**  **No. of Methylated Molec/100µl** |
| 18_1 | 2/26/92 | 2/25/92 | Postoperative | 0 | 18 | 5 |
| 18_2 | 2/27/92 |  |  | 0 | 9 | 22 |
| 18_3 | 4/8/92 |  |  | 6 | 4 | 3 |
| 18_4 | 5/6/92 |  |  | 10 | 6 | 2 |
| 18_5 | 6/3/92 |  |  | 14 | 5 | 0 |
| 18_6 | 6/30/92 |  |  | 18 | 11 | 2 |
| 18_7 | 7/28/92 |  |  | 22 | 6 | 1 |
| 18_8 | 8/11/92 |  |  | 24 | 9 | 0 |
| 18_9 | 9/3/92 |  |  | 27 | 9 | 3 |
| 18_10 | 10/1/92 |  |  | 31 | NA | 1 |
| 18_11 | 11/16/92 |  |  | 38 | 5 | 1 |
| 18_12 | 6/28/93 |  |  | 70 | 5 | 2 |
| 18_13 | 2/3/94 |  |  | 101 | 7 | 1 |
| 18_14 | 3/24/94 |  |  | 108 | 6 | 0 |
| 18_15 | 7/13/94 |  |  | 124 | 2 | 1 |
| 18_16 | 4/5/95 |  |  | 162 | 4 | 8 |
| 18_17 | 10/4/95 |  |  | 188 | 3 | 1 |
| 18_18 | 11/22/95 |  |  | 195 | 3 | 2 |
| 18_19 | 12/4/95 |  |  | 197 | 4 | 2 |
| 18_20 | 11/16/96 |  |  | 246 | 8 | 32 |

**Supplemental Table 4 (continued)**

| **Patient 19** | | | | | | |
| --- | --- | --- | --- | --- | --- | --- |
| **Sample ID** | **Collection Date** | **Surgery Date** | **Baseline Sample** | **Weeks of Follow-up** | **CA-125* Levels (U/ml)** | **IFFO1-M**  **No. of Methylated Molec/100µl** |
| 19_1 | 9/18/1997 | 8/19/97 | Postoperative | 0 | 884 | 3 |
| 19_2 | 10/9/1997 |  |  | 3 | 75 |  |
| 19_3 | 10/30/1997 |  |  | 6 | 26 |  |
| 19_4 | 11/21/1997 |  |  | 9 | 13 |  |
| 19_5 | 12/11/1997 |  |  | 12 | 9 |  |
| 19_6 | 1/7/1998 |  |  | 16 | 6 |  |
| 19_7 | 2/26/1998 |  |  | 23 | 6 |  |
| 19_8 | 11/12/1998 |  |  | 60 | 42 |  |
| 19_9 | 12/15/1998 |  |  | 65 | 46 |  |
| 19_10 | 2/18/1999 |  |  | 74 | 51 |  |
| 19_11 | 6/16/1999 |  |  | 91 | 89 |  |
| 19_12 | 7/15/1999 |  |  | 95 | 116 |  |

| **Patient 21** | | | | | | |
| --- | --- | --- | --- | --- | --- | --- |
| **Sample ID** | **Collection Date** | **Surgery Date** | **Baseline Sample** | **Weeks of Follow-up** | **CA-125* Levels (U/ml)** | **IFFO1-M**  **No. of Methylated Molec/100µl** |
| 21_1 | 11/4/99 | 10/25/99 | Postoperative | 0 | 274 | 17 |
| 21_2 | 11/30/99 |  |  | 4 | 105 | 1 |
| 21_3 | 12/20/99 |  |  | 7 | 33 | 6 |
| 21_4 | 1/11/00 |  |  | 10 | 13 | 1 |
| 21_5 | 1/20/00 |  |  | 11 | 25 | 4 |
| 21_6 | 2/10/00 |  |  | 14 | 11 | 1 |
| 21_7 | 3/2/00 |  |  | 17 | 6 | 1 |
| 21_8 | 3/23/00 |  |  | 20 | 9 | 3 |
| 21_9 | 4/12/00 |  |  | 23 | 7 | 3 |
| 21_10 | 4/19/00 |  |  | 24 | 10 | 0 |
| 21_11 | 5/10/00 |  |  | 27 | 9 | 3 |
| 21_12 | 7/20/00 |  |  | 37 | 166 | 3 |
| 21_13 | 8/8/00 |  |  | 40 | 410 | 3 |

NA = not available

***** Interpretation of serum CA-125 levels have been based on a normal value of less than 35 U/ml
